# How Specific is Alcohol-Specific Self-Control? A Longitudinal Study of the Mediating Role of Alcohol-Specific Self-Control in the Relation Between General Self-Control and Adolescent Alcohol Use

**DOI:** 10.1007/s10935-023-00737-z

**Published:** 2023-06-28

**Authors:** Suzanne M. Geurts, Ina M. Koning, Catrin Finkenauer

**Affiliations:** Department of Interdisciplinary Social Science, Utrecht University, Padualaan 14, Utrecht, 3584 CH The Netherlands

**Keywords:** adolescents, alcohol use, general self-control, alcohol-specific self-control, domain-specific

## Abstract

Although accumulating studies indicate that alcohol-specific self-control can be useful in predicting adolescent alcohol use, little is known about its specificity. This longitudinal study aimed to advance our understanding of domain-specific self-control by examining whether alcohol-specific self-control mediates the effect of general self-control on adolescent alcohol use or has generalizing effects by also mediating the effect of general self-control on other behavior requiring self-control (adolescent digital media use and smoking). Data from 906 adolescents aged 11–14 years who were enrolled in the Dutch study Prevention of Alcohol Use in Students were used. Data were collected using online questionnaires at four annual measurements. Structural equation modelling revealed that higher alcohol-specific self-control fully mediated the effect of higher general self-control on alcohol use. Alcohol-specific self-control did not mediate the effect of higher general self-control on digital media use, but did partially mediate the effect of higher general self-control on smoking. These results suggest that alcohol-specific self-control is domain-specific, but not necessarily substance-specific. The domain-specificity of alcohol-specific self-control provides evidence for its theoretical relevance for the explanation of adolescent alcohol use. It also suggests leverage points for intervention programs focusing on improving alcohol-specific self-control to reduce adolescent alcohol use.

## Background

One of the most commonly used drugs among adolescents is alcohol. In 2021, 47.5% of Dutch adolescents aged 12–16 years had consumed alcohol at least once in their life, 27.8% at least once in the past month and 17.7% had been drunk or tipsy at least once in their life. Among 16-year-olds, these percentages are notably higher; respectively 76%, 59.9% and 46.6% (Boer et al., 2022). Adolescent alcohol use is associated with alcohol problems in adulthood (DeWit et al., 2000; Guttmannova et al., 2011; Marshall, 2014) and problem behavior in middle and high school, such as early school leaving, violent and delinquent behavior, sexual risk behavior and co-morbid substance use (Ellickson et al., 2003; Green et al., 2016; Kebede et al., 2005; Hingson & Zha, 2018; Jones et al., 2019; Komro et al., 2010; Rocca et al., 2019; Soares et al., 2019). Additionally, drinking at an early age can have damaging effects on adolescent brain development, especially on learning and attention abilities (Bava & Tapert, 2010; Lees et al., 2020; Spear, 2018). Because of these negative outcomes, it is important to prevent and reduce adolescent alcohol use. Since self-control has a direct influence on adolescent alcohol use (e.g., Griffin et al., 2000; Koning et al., 2011) and given the possibility to improve this individual characteristic (Ridder et al., 2020; Strayhorn, 2002), alcohol-specific self-control may be an important factor to target in preventing and reducing adolescent alcohol use.

Self-control is considered an individual characteristic that reflects the ability to control and adjust thoughts, emotions and behavioral tendencies, and refrain from acting on them in order to achieve a goal or conform to standards (Ridder et al., 2012; D’Lima et al., 2012). It includes capacities such as emotion regulation, thought suppression, temptation resistance and behavior modification (De Ridder et al., 2012). A theory that identifies self-control as the key concept in understanding risk behavior among adolescents is the self-control theory by Gottfredson and Hirschi (1990). This theory assumes that individuals with low levels of self-control are more likely to engage in risk behavior, such as alcohol use, because they have a greater likelihood to react to stimuli in the environment that make them overstep their norms (Visser et al., 2013). Particularly in adolescence, high self-control can play an important role in preventing engagement in and reducing risk behavior (Crone & Dahl, 2012; Jia et al., 2021; Tangney et al., 2004; Tian et al., 2022).

In the literature a distinction is made between general self-control, which is assumed to affect behaviors across different domains (De Ridder et al., 2012; Tsukayama et al., 2011), and domain-specific self-control, such as alcohol-specific self-control, which refers to the ability to refrain from drinking alcohol (Blalock et al., 2022; Jang et al., 2013; Lindgren et al., 2014; Oei & Burrow, 2000; Venables et al., 2018). A number of studies suggest that both general self-control and alcohol-specific self-control are related to adolescent alcohol use (e.g., Bogg et al., 2012; Lindgren et al., 2014; Yeh et al., 2005). However, to our knowledge, it has not yet been investigated whether the relation between general self-control and adolescent alcohol use is mediated by alcohol-specific self-control, and whether this mediating role is domain- and substance-specific.

### General Self-Control and Adolescent Alcohol Use

A wide range of empirical studies examined the relation between general self-control and adolescent alcohol use and revealed that high general self-control is an important protective factor for several alcohol-related outcomes (Bogg et al., 2012; Bräker et al., 2015; Copeland et al., 2020; Cheung, 2014; Davies et al., 2017; Griffin et al., 2000; Innamorati and Maniglio, 2015; Koning et al., 2014; Kwon, 2013; Lindgren et al., 2014; Hoffman, 2022; Piquero et al., 2002; Quinn and Fromme, 2010; Vazsonyi et al., 2006). Longitudinal studies have demonstrated the effects of general self-control on adolescents’ alcohol use. For example, Kwon (2013) found that the level of general self-control at age 11 predicted the frequency of alcohol use at age 15. Moreover, in a sample of Dutch adolescents, higher general self-control at age 13 predicted a lower frequency and quantity of alcohol consumption one year later (Koning et al., 2014). Hence, the literature converges to suggest that a higher level of self-control is associated with lower levels of alcohol use among adolescents.

### Alcohol-Specific Self-Control and Adolescent Alcohol Use

Next to general self-control, previous research also provides evidence for a relation between alcohol-specific self-control and adolescent alcohol use. Though most studies were conducted among college students (e.g., Ehret et al., 2013; Miller et al., 2019; Oei and Jardim, 2007; Plata et al., 2022), among high school students too higher alcohol-specific self-control was associated with less alcohol use (Jang et al., 2013; Lee et al., 2020; Yeh et al., 2005). Moreover, a longitudinal study found that alcohol-specific self-control predicted the frequency and quantity of drinking among high school students one year later (Connor et al., 2011). Thus, although longitudinal evidence among high school students is scarce, existing findings indicate that a higher level of alcohol-specific self-control is associated with less adolescent alcohol use.

### General Self-Control, Alcohol-Specific Self-Control and Adolescent Alcohol Use

Though both general and alcohol-specific self-control relate to adolescents’ drinking, alcohol-specific self-control may be a proximal factor that accounts for the more distal influence of general self-control on adolescent alcohol use. To our knowledge, the mediating role of alcohol-specific self-control in the relation between general self-control and adolescent alcohol use has not yet been examined. However, Lindgren et al. (2014) investigated the unique influence of both general and alcohol-specific self-control on multiple alcohol outcomes among college students. Both types of self-control were related to more favorable outcomes, but the association with alcohol-specific self-control was stronger than the association with general self-control. This indicates that alcohol-specific self-control may be a more proximal factor and general self-control a more distal factor of alcohol use. Additionally, Lindgren and colleagues found that general self-control was positively related to alcohol-specific self-control (Lindgren et al., 2014). However, due to the cross-sectional design of their study, no conclusion could be drawn about the direction of the effect. Adolescents with higher general self-control have less difficulty in resisting temptations in general, because they are better able to foresee the long-term costs of their behavior (Burt et al., 2020; Ridder et al., 2012). Thus, we expect that adolescents with higher general self-control have higher alcohol-specific self-control, because they can resist the temptation of drinking, which in turn, leads to less alcohol consumption (Connor et al., 2011). So, we hypothesize that the effect of general self-control on adolescent alcohol use can (partially) be explained by alcohol-specific self-control.

### Specificity of Alcohol-Specific Self-Control

Little is known about the extent to which alcohol-specific self-control is actually domain- and substance-specific. Domain-specificity implies that alcohol-specific self-control is specifically related to the domain of substance use and not to other domains (e.g., digital media use; Tsukayama et al., 2011). Substance-specificity implies that alcohol-specific self-control only relates to alcohol use and not to other substance use. One study examined whether alcohol-specific self-control is related to alcohol use and other behaviors including smoking, caffeine consumption and exercise behavior (Oei & Burrow, 2000). The findings showed that alcohol-specific self-control did not predict these other behaviors, but it did predict the quantity of alcohol consumption. This supports the idea that alcohol-specific self-control is domain- as well as substance-specific. However, cross-sectional data were used and the participants in the study were first-year psychology students (mean age 20 years; Oei and Burrow, 2000). Therefore, it is not possible to draw conclusions about the direction of the effect and the findings may not generalize to adolescents in general. Based on the existing study, it is expected that alcohol-specific self-control is specifically related to adolescent alcohol use and not to other behaviors.

### Current Study

In this 4-wave study among 906 adolescents aged 11–14 years, we aimed to examine whether the mediating role of alcohol-specific self-control in the relation between general self-control and adolescent alcohol use is domain- and substance-specific. The answer to this question has important implications for the use of alcohol-specific self-control in the explanation of adolescent alcohol use and for the content and efficiency of substance-related interventions. To answer this question, it is necessary to investigate whether this mediation effect only applies for the effect of general self-control on adolescent alcohol use, or also for the effect of general self-control on other behaviour that is considered a temptation for adolescents to engage in, and therefore requires self-control (to test domain-specificity), and on other substance use (to test substance-specificity). Therefore, we included adolescent digital media use (Schulz van Endert, 2021; Twenge and Campbell, 2018) and smoking (Astolfi et al., 2021) as control outcome measures. Based on the self-control theory and previous empirical research, we hypothesized that (1) the effect of a higher level of general self-control on less adolescent alcohol use is mediated by a higher level of alcohol-specific self-control, and that (2) the mediating role of alcohol-specific self-control in the effect of general self-control on adolescent alcohol use is only related to adolescent alcohol use and not to adolescent digital media use and smoking.

## Methods

### Procedure

The longitudinal data used in this study are part of a cluster randomized trial of a Dutch alcohol prevention program for adolescents called Prevention of Alcohol Use in Students (PAS; Koning et al., 2009). In this randomized controlled trial study, 80 schools were randomly selected from a list of all public secondary schools in the Netherlands and invited to participate. Schools were randomly assigned to one of the four conditions: three intervention conditions and one control condition which did not receive any intervention. In the current study, only data from the control condition were used to ensure that the data were not affected by possible intervention effects. All 7th grade students were involved in the study. Parents of the students received a letter of consent in order to give them the possibility to refuse their children’s participation (0.01% refusal). The data were collected in 2006 (T1), 2007 (T2), 2008 (T3) and 2009 (T4) in classrooms during school time through a questionnaire that was available on a secured website. The study protocol was assessed and approved by the Medical Ethical Committee. Data can be requested by contacting the corresponding author.

### Participants

At T1, 906 adolescents between the age of 11 and 14 years (*M* = 12.19, *SD* = 0.51) from four different secondary schools participated in the study. Of these adolescents, 52.5% were boys and 95.5% were born in the Netherlands. Sixty comma 2% enrolled in pre-vocational education (lowest levels that give option to continue in vocational education) and 39.7% in general and pre-university education (highest levels that give the option to continue in tertiary higher education). Religious background of the adolescents was primarily non-religious (46.1%) and Roman Catholic (34.5%). A total of 865 adolescents (95.47%) at T2, 802 adolescents (88.52%) at T3 and 778 adolescents (85.87%) at T4 completed the follow-up assessments. Reasons for attrition were no permission or adolescents were not present at the day of data collection. Attrition analyses showed no differences at T1 between adolescents who completed the follow-up assessment at T2 and adolescents who did not with respect to gender, age, educational level, general self-control and alcohol use. Nonresponding adolescents at T3 and T4 significantly differed from responding adolescents at these follow-up assessments in being older (T3: *t*(121) = -2.26, *p* = .025; T4: *t*(159) = -3.09, *p* = .002), being in lower education (T3: *F*(1,904) = 19.09, *p* < .001; T4: *F*(1, 904) = 20.26, *p* < .001), having lower general self-control (T3: *t*(901) = 2.87, *p* = .004; T4: *t*(901) = 2.38, *p* = .018), and smoking more frequently at T1 (T3: *t*(108) = -2.41, *p* = .018; T4: *t*(132) = -3.11, *p* = .002). No differences were found for gender and alcohol use at T1, and alcohol-specific self-control at T2.

### Measures

*Alcohol use* was the outcome variable in this study. By using the Quantity-Frequency measure, we computed the average number of glasses consumed in a week (Koning et al., 2009). We asked adolescents how many days they usually consume alcohol during the week (Monday to Thursday) and during the weekend (Friday to Sunday) to assess the frequency of alcohol use. We measured the quantity of alcohol use by asking how many glasses of alcohol the adolescent usually drinks on a weekday and on a weekend day. Thereafter, we computed the average weekly alcohol use by calculating the product of the number of days and the number of glasses for weekdays and weekend days separately, after which these two products were added up. Higher scores indicated higher levels of alcohol use.

*Digital media use* was one of the control outcome variables and was measured by asking how many hours adolescents spent doing the following activities on a weekday and on a weekend day: watching television (films and DVD’s included), playing computer games or video games (PlayStation, Xbox, GameCube, etc.) and using the Internet for chatting, messaging, surfing the Web, etc. Response options ranged from 1 (*never*) to 9 (*7 h or more*). The sum of the 6 items were computed. Higher scores reflected higher levels of digital media use.

*Smoking* was the other control outcome variable and was measured by asking the question: “How often do you currently smoke?”. Possible responses were 1 (*I do not smoke*), 2 (*less than once a week*), 3 (*at least once a week, but not every day*), 4 (*every day*). Higher scores indicated more frequent smoking.

*General self-control* is the ability to control and adjust thoughts, emotions and behaviors (De Ridder et al., 2012). It was measured by using the brief Self-Control Scale developed and tested by Tangney et al. (2004) which consists of 13 items. Response options ranged from 1 (*never*) to 5 (*very often*). Example items are “I am good at resisting temptation”, “I often act without thinking through all the alternatives” and “I say inappropriate things”. The mean score of the 13 items was computed. Several items were reversely scored, so that higher scores reflected higher general self-control. Cronbach’s alpha was 0.72 at T1 and 0.77 at T2.

*Alcohol-specific self-control* refers to the ability to refrain from drinking alcohol (Jang et al., 2013) and was measured by two items: “Are you able to resist drinking alcohol when your friends are offering you a glass of alcohol?” and “Are you able to resist drinking alcohol when all your friends are drinking?”. The mean score of these two items rated on a 4-point scale from 1 (*definitely not*) to 4 (*definitely*) was used, with higher scores indicating higher alcohol-specific self-control. Pearson correlation was 0.80 at T2 and 0.83 at T3.

*Gender, age and educational level* were included as control variables, because these factors are related to alcohol use (Johnson et al., 2010; Van Dorsselaer et al., 2016; Webb et al., 2002). Adolescents reported their gender (*boy* = 0, *girl* = 1) and date of birth. Educational level was known, because the data were collected at schools and adolescents were in classes representing a specific level of education (*lower secondary vocational education* = 0, *higher general secondary and pre-university education* = 1).

### Data Analysis

Missing data were estimated in Mplus using the Full Information Maximum Likelihood with Robust Standard Errors default setting, allowing information of all participants to be used for analysis (Muthén & Muthén, 2015; Werner, 2000). Descriptive statistics and correlations were obtained. We used structural equation modeling to test the effect of general self-control on adolescent alcohol use, digital media use and smoking and the extent to which these associations are mediated by alcohol-specific self-control. In all models, we controlled for gender, age, level of education and outcome at previous wave.

**Fig. 1:**
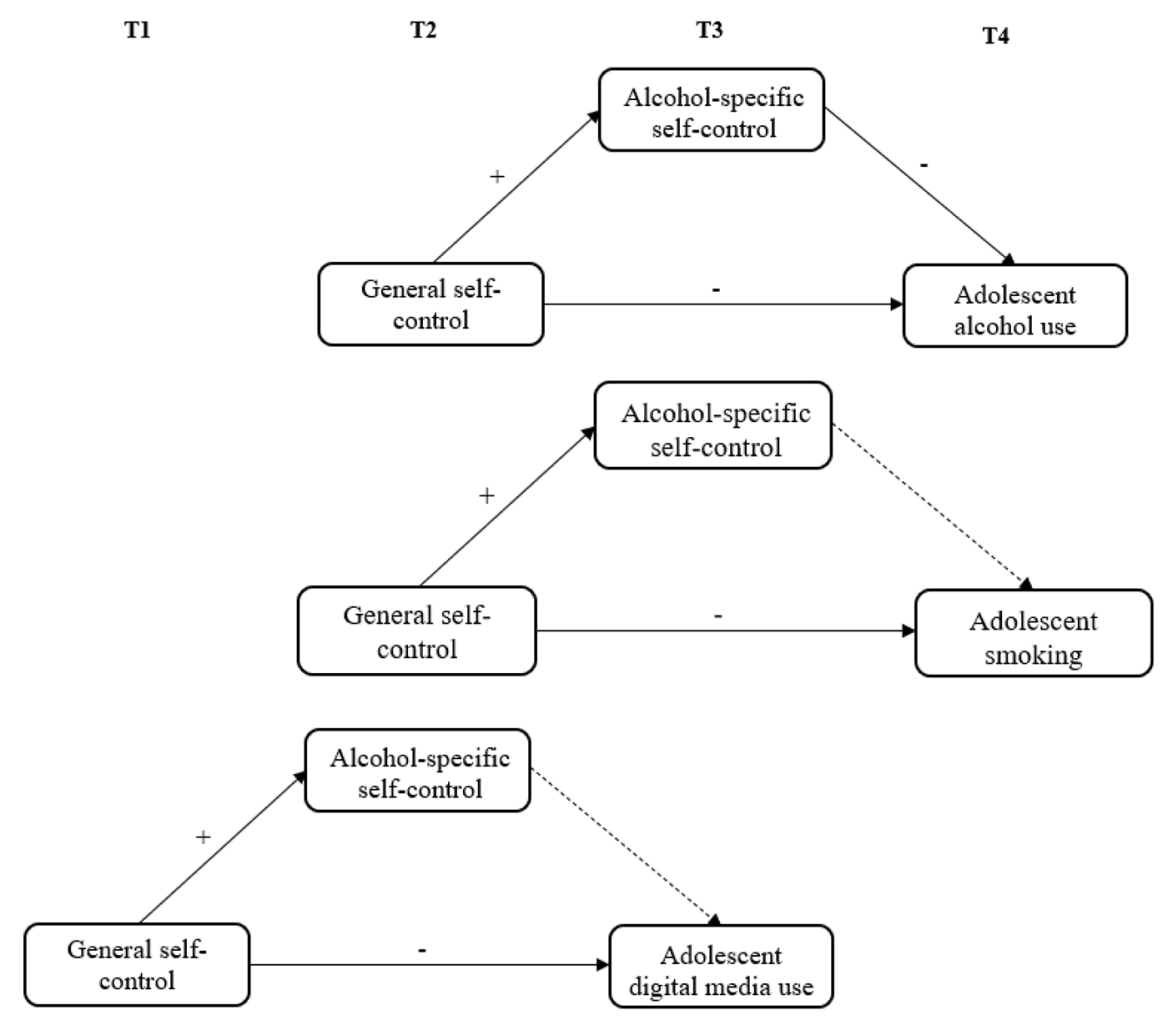
Research model

Alcohol-specific self-control was not included in the questionnaire at T1 and adolescent digital media use was not included in the questionnaire at T1 and T4. Therefore, the following measurement moments were used for the different analyses. First, the direct effect of general self-control at T2 on adolescent alcohol use/smoking at T4 was tested. Second, the effect of general self-control at T2 on the mediating variable alcohol-specific self-control at T3 was analyzed. Third, the effect of alcohol-specific self-control at T3 on adolescent alcohol usesmoking at T4 was tested while controlling for the effect of general self-control at T2. Finally, the model indirect command in Mplus was used to examine whether the mediated effect was statistically significant. In the analyses with digital media use as outcome measure, general self-control at T1, alcohol-specific self-control at T2 and adolescent digital media use at T3 were used. Adolescent digital media use at T2 was included as control variable. Figure 1 depicts a graphical representation of the research model. The MLR estimator is robust to non-normality of the outcome variables and is therefore used in the analysis. In line with Nieminen et al. (2013), we used the standardized βs as effect size indices, whereby β < 0.2 was considered a small, 0.2 < β < 0.5 a moderate, and β > 0.5 a strong effect.

## Results

### Descriptive Results

Table 1 depicts all descriptive data. The average amount of alcohol adolescents consumed at T2 was 1.9 glasses per week (*SD* = 8.43). At T4 the average amount was substantially higher: 6 glasses per week (*SD* = 13.10). On the contrary, general self-control and alcohol-specific self-control were quite stable over time; general self-control: *M* = 3.6 (*SD* = 0.51) at T1 and *M* = 3.5 (*SD* = 0.57) at T2; alcohol-specific self-control: *M* = 3.2 (*SD* = 0.88) at T2 and *M* = 3.2 (*SD* = 0.91) at T3. Adolescents spent, on average, 21.2 h (*SD* = 10.05) in total per week watching television, playing computer games or video games and using the Internet at T2 and 21.8 h (*SD* = 10.29) at T3. With regard to smoking, at T2 89.9% of the adolescents reported not smoking, 5.1% at least once a week, but not every day, 2.2% less than once a week and 2,8% reported to smoke every day. At T4 these percentages were 77%, 5.8%, 5.6% and 11.5% respectively.

**Table 1:** Descriptive Statistics of Research Variables

	*n*(%)	*M*(SD)	Min	Max
Gender (boys)	476(52.5%)			
Educational level (lower)	546(60.3%)			
Age T1		12.2(0.51)	11	14
General self-control T1		3.6(0.51)	2	4.92
General self-control T2		3.5(0.57)	1.23	5
Alcohol-specific self-control T2		3.2(0.88)	1	4
Alcohol-specific self-control T3		3.2(0.91)	1	4
Alcohol use T2		1.9(8.43)	0	104
Alcohol use T4		6.0(13.10)	0	104
Digital media use T2		21.2(10.05)	0	48
Digital media use T3		21.8(10.29)	0	48
SmokingT2		1.2(0.60)	1	4
SmokingT4		1.5(1.03)	1	4

*Note. n* = number of participants; *M* = mean; *SD* = standard deviation

Table 2 presents the Pearson correlations between all variables of interest. Both types of self-control were significantly negatively correlated with alcohol use, digital media use and smoking, indicating that higher general and alcohol-specific self-control were related to less drinking, digital media use and smoking. In addition, a higher level of general self-control was significantly related to a higher level of alcohol-specific self-control.

**Table 2:** Correlations between Alcohol Use, Digital Media Use, Smoking, Alcohol-Specific Self-Control, General Self-Control and Control Variables

Variable	1	2	3	4	5	6	7	8	9	10	11	12	13
1. Gender (ref = boys)	1.00												
2. Age	− 0.05	1.00											
3. Educational level (ref = lower)	− 0.03	− 0.23**	1.00										
4. General self-control T1	0.04	− 0.08*	0.17**	1.00									
5. General self-control T2	0.08*	− 0.07*	0.13**	0.54**	1.00								
6. Alcohol-specific self- control T2	0.06	− 0.01	0.08*	0.20**	0.36**	1.00							
7. Alcohol-specific self-control T3	− 0.00	− 0.02	0.09*	0.17**	0.30**	0.37**	1.00						
8. Alcohol use T2	− 0.08*	0.13**	− 0.14**	− 0.18**	− 0.29**	− 0.30**	− 0.20**	1.00					
9. Alcohol use T4	− 0.17*	0.07	− 0.09*	− 0.15**	− 0.21**	− 0.27**	− 0.35**	0.42**	1.00				
10. Digital media use T2	− 0.12**	0.03	− 0.16**	− 0.20**	− 0.23**	− 0.13**	− 0.09*	0.20**	0.16**	1.00			
11. Digital media use T3	− 0.15**	0.02	− 0.17**	− 0.18**	− 0.18**	− 0.09**	− 0.09**	0.13**	0.19**	0.44**	1.00		
12. Smoking T2	0.00	0.07*	− 0.11**	− 0.23**	− 0.28**	− 0.23**	− 0.20**	0.46**	0.27**	0.16**	0.08*	1.00	
13. Smoking T4	0.02	0.03	− 0.21**	− 0.22**	− 0.22**	− 0.18**	− 0.20**	0.37**	0.40**	0.16**	0.16**	0.41**	1.00

*Note.* Spearman correlation was used for ordinal and continuous variables. Pearson Correlation was used for dichotomous variables

* *p* < .05. ** *p* < .01

### Mediating Role of Alcohol-Specific Self-Control on Adolescent Alcohol Use

First, we tested the direct effect of general self-control at T2 on alcohol use at T4. Results showed that general self-control significantly predicted future alcohol use while controlling for gender, age, educational level and adolescent alcohol use at T2 (see Table 3). Adolescents who reported higher levels of general self-control consumed less alcohol on a weekly basis two years later (*β* = -0.101, *p* = .028). The model explained 16.8% of the variance in adolescent alcohol use.

**Table 3:** Structural Equation Model of the Effects of General Self-Control and Alcohol-Specific Self-Control on Adolescent Alcohol Use

	Step 1 (X on Y): Alcohol use T4		Step 2 (X on M): Alcohol-specific self-control T3		Step 3 (X on Y via M): Alcohol use T4	
Variable	*β*	*SE*	*β*	*SE*	*β*	*SE*
Gender (ref = boys)	-0.14***	0.03	-0.03	0.03	-0.15***	0.03
Age	0.03	0.04	-0.01	0.04	0.03	0.04
Educational level (ref = lower)	-0.03	0.03	0.03	0.03	-0.03	0.03
Alcohol use T2	0.32*	0.13			0.29*	0.13
Alcohol-specific						
self-control T2			0.29***	0.05		
General self-control						
T2	-0.10*	0.05	0.23***	0.04	-0.04	0.04
Alcohol-specific						
self-control T3					-0.20***	0.05

*Note. β* = standardized coefficient; *SE* = standard error

* *p* < .05. ** *p* < .01. *** *p* < .001

To test the indirect effect of general self-control on adolescent alcohol use via alcohol-specific self-control, we examined the effect of general self-control at T2 on alcohol-specific self-control at T3. As shown in Table 3; Fig. 1, general self-control was a significant predictor of alcohol-specific self-control while controlling for gender, age, educational level and alcohol-specific self-control at T2. Higher levels of general self-control predicted higher levels of alcohol-specific self-control (*β* = 0.226, *p* < .001). The model explained 18.8% of the variance in alcohol-specific self-control.

Next, we tested the full mediation model (Table 3; Fig. 1). According to Hu and Bentler’s (1999) cut-off criteria for fit indexes to evaluate model fit, the mediation model showed a good fit to the data: CFI = 0.99, RMSEA = 0.031, [χ]² = 3.762(2), *p* = .152. We found a significant negative effect of alcohol-specific self-control at T3 on alcohol use at T4 while controlling for the effect of general self-control at T2 (*β* = -0.200, *p* < .001). In this step, the effect of general self-control at T2 on alcohol use at T4 was no longer significant (*β* = -0.043, *p* = .297). A significant total indirect effect of general self-control on use via alcohol-specific self-control was found, indirect = -0.045, *p* = .001). The model explained 19.4% of the variance in adolescent alcohol use.

### Mediating Role of Alcohol-Specific Self-Control on Adolescent Digital Media Use

As Table 4 shows, a significant negative effect of general self-control at T1 on digital media use at T3 was found. That is, adolescents with higher general self-control reported less digital media use two years later (*β* = -0.089, *p* = .012). The model explained 22% of the variance in adolescent digital media use.

**Table 4:** Structural Equation Model of the Effects of General Self-Control and Alcohol-Specific Self-Control on Adolescent Digital Media Use

	Step 1 (X on Y): Digital media use T3		Step 2 (X on M): Alcohol-specific self-control T2		Step 3 (X on Y via M): Digital media use T3	
Variable	*β*	*SE*	*β*	*SE*	*β*	*SE*
Gender (ref = boys)	-0.12***	0.03	0.05	0.03	-0.12***	0.03
Age	-0.06	0.03	0.01	0.04	-0.06	0.03
Educational level (ref = lower)	-0.11***	0.03	0.05	0.03	-0.11***	0.03
Digital media use T2	0.38***	0.04			0.38***	0.04
General self-control						
T1	-0.09*	0.04	0.20***	0.03	-0.09*	0.04
Alcohol-specific						
self-control T2					-0.02	0.04

*Note. β* = standardized coefficient; *SE* = standard error

* *p* < .05. ** *p* < .01. *** *p* ≤ .001

We examined the effect of general self-control at T1 on alcohol-specific self-control at T2 (Table 4). A higher level of general self-control at T1 significantly predicted a higher level of alcohol-specific self-control at T2 while controlling for age, gender and educational level (*β* = 0.201, *p* < .001). The model explained 4.8% of the variance in alcohol-specific self-control.

The full mediation model showed an acceptable/good fit to the data, CFI = 0.96, RMSEA = 0.098, [χ]² = 9.694(1), *p* < .05. We found no significant effect of alcohol-specific self-control at T2 on digital media use at T3 (*β* = -0.016, *p* = .686). Also, the model indirect command showed no significant indirect effect of general self-control on digital media use via alcohol-specific self-control, indirect = -0.003, *p* = .693) (see Table 4). The model explained 21.9% of the variance in adolescent digital media use.

### Mediating Role of Alcohol-Specific Self-Control on Adolescent Smoking

Analyses showed a significant negative effect of general self-control at T2 on smoking at T4 while controlling for gender, age, educational level and adolescent smoking at T2 (see Table 5). Adolescents with higher general self-control smoked less two years later (*β* = -0.137, *p* = .001). The model explained 27% of the variance in adolescent smoking.

**Table 5:** Structural Equation Model of the Effects of General Self-Control and Alcohol-Specific Self-Control on Adolescent Smoking

	Step 1 (X on Y): Smoking T4		Step 2 (X on M): Alcohol-specific self-control T3		Step 3 (X on Y via M): Smoking T4	
Variable	*β*	*SE*	*β*	*SE*	*β*	*SE*
Gender (ref = boys)	0.03	0.03	-0.03	0.03	-0.03	0.03
Age	0.00	0.04	-0.01	0.04	0.01	0.04
Educational level (ref = lower)	-0.14***	0.03	0.03	0.03	-0.13***	0.03
Smoking T2	0.42***	0.05			0.41***	0.05
Alcohol-specific						
self-control T2			0.29***	0.05		
General self-control						
T2	-0.14**	0.04	0.23***	0.04	-0.11*	0.04
Alcohol-specific						
self-control T3					-0.10*	0.04

*Note. β* = standardized coefficient; *SE* = standard error

* *p* < .05. ** *p* < .01. *** *p* < .001

**Fig. 2:**
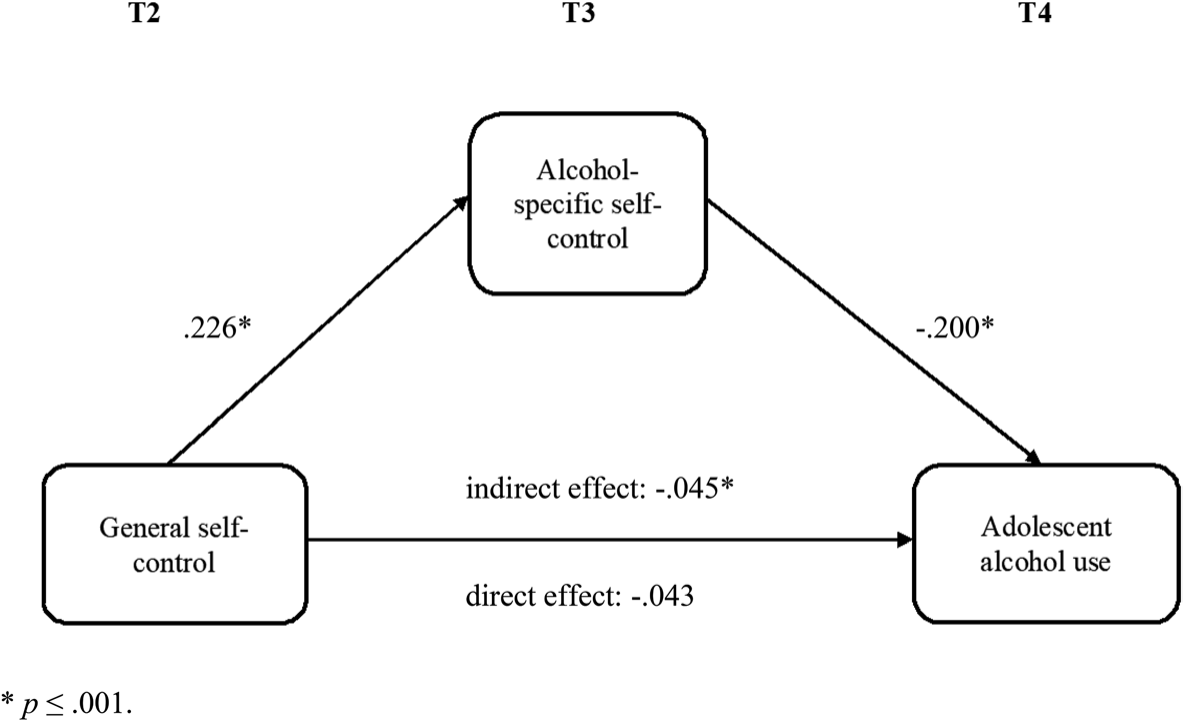
Structural equation model of the effect of general self-control on adolescent alcohol use via alcohol-specific self-control

The mediation effect of general self-control on smoking through alcohol-specific self-control was tested next. The mediation model showed a good fit to the data: CFI = 0.98, RMSEA = 0.063, [χ]² = 9.163(2), *p* = .010. As shown in Table 5; Fig. 2, we found a significant negative effect of alcohol-specific self-control at T3 on smoking at T4 while controlling for the effect of general self-control at T2 (*β* = -0.104, *p* = .014). The effect of general self-control at T2 on smoking at T4 was still significant (*β* = -0.107, *p* = .013). The model indirect command showed a significant total indirect effect of general self-control on smoking via alcohol-specific self-control, indirect = -0.023, *p* = .034). The model explained 27% of the variance in adolescent smoking.

## Discussion

As far as we know, this is the first study that longitudinally investigated whether the effect of general self-control on adolescent alcohol use is mediated by alcohol-specific self-control and to what extent this mediating role is domain- and substance-specific. In order to answer the question whether the role of alcohol-specific self-control is domain- and substance-specific, we examined if its mediating effect only applies to the effect of general self-control on adolescent alcohol use or extends to the effect of general self-control on adolescent digital media use and/or smoking. Results from a longitudinal study among a large group of adolescents showed that the effect of general self-control on alcohol use was fully mediated by alcohol-specific self-control. Furthermore, the effect of general self-control on smoking was partially mediated by alcohol-specific self-control, but alcohol-specific self-control did not mediate the effect of general self-control on digital media use. These results imply that general self-control predicts alcohol-specific self-control, which seems to be domain-specific, but not substance-specific.

Consistent with previous research (e.g., Copeland et al., 2020; Cheung, 2014; Hoffman, 2022; Kwon, 2013; Nakhaie et al., 2000; Quinn and Fromme, 2010) and the self-control theory (Gottfredson & Hirschi, 1990), high general self-control seemed to be a protective factor of adolescent risk behavior, because it predicted less alcohol use, smoking and digital media use in adolescence two years later. Consistent with our first hypothesis, the effect of general self-control on alcohol use was completely explained by the level of alcohol-specific self-control. That is, adolescents with a greater ability to control and adjust thoughts, emotions and behavioral tendencies in general were more able to resist the temptations associated with alcohol use, which in turn predicted less alcohol consumption. The fact that alcohol-specific self-control fully mediated the effect of general self-control on alcohol use indicates that alcohol-specific self-control is a more proximal predictor of weekly alcohol use of adolescents than general self-control. Our findings contribute to Lindgren et al. (2014) by showing that the association between alcohol consumption and alcohol-specific self-control was stronger than the association between alcohol consumption and general self-control. Possibly that the role of general self-control in adolescents’ alcohol use is dependent on whether it concerns the level of alcohol use or the onset of alcohol use. Some research suggests that general self-control might be a better predictor for the onset of adolescent alcohol use than alcohol-specific self-control. Tipton and Worthington (1984) suggested that domain-specific self-control is a better predictor of an individual’s behavior in a clearly defined and familiar situation, and that general self-control is a better predictor of someone’s behavior in situations which are more ambiguous and less familiar to the individual. Adolescents’ first time exposure to peers who use substances may be an example of a more ambiguous and unfamiliar situation. Research shows that alcohol onset is associated with exposure to peers who use alcohol (Cook et al., 2023; Dupre et al., 1995; Kosterman et al., 2000; Mundt, 2011; Yuen et al., 2020). Adolescents who already drink may be more familiar with situations in which their friends are drinking or in which they are offered a drink by a friend. Therefore, it is possible that alcohol-specific self-control is a better predictor of the level of adolescent alcohol use and general self-control a better predictor of the onset of adolescent alcohol use. Our finding that alcohol-specific self-control is a more proximal predictor of the amount of adolescent alcohol use than general self-control supports this suggestion. However, it remains for future studies to examine whether alcohol-specific self-control also acts like a mediator of the effect of general self-control on the onset of alcohol use.

Our results partly confirmed our second hypothesis. Alcohol-specific self-control did not mediate the effect of higher general self-control on less digital media use, but did partially mediate the effect of higher general self-control on less smoking. This provides evidence that alcohol-specific self-control is domain-specific in that it affected substance use; alcohol use as well as cigarette use. This is in contrast with Oei and Burrow (2000) who found in their cross-sectional study that alcohol-specific self-control is specifically related to alcohol use and not to other substance use. An explanation for our finding that the mediating role of alcohol-specific self-control extended to smoking is that drinking alcohol and smoking are relatively strongly correlated (*r*_s_ =0.46 at T2 and *r*_s_=0.40 at T4). The link between alcohol use and smoking among adolescents has been shown in various studies (e.g., Ball et al., 2023; Duncan et al., 1998; Jackson et al., 2002; Keetile et al., 2023; Lim et al., 2023). Based on our findings, strengthening adolescents’ ability to refrain from drinking alcohol does not have beneficial effects on their ability to resist temptations in other domains (e.g., digital media use), but it may affect their ability to resist other substances the use of which is strongly associated with alcohol use, such as cigarettes. This suggests that it is possible for adolescents to transfer their self-control skills to resist alcohol to situations which involve temptations of other substances. Nevertheless, it remains to be examined whether it is more effective to improve self-control skills for substances in general or to focus on skills addressing each specific substance separately.

Since the results of the current study showed that the ability to refrain from drinking alcohol plays an important role in predicting the quantity of adolescent alcohol use, it is important to know which factors affect alcohol-specific self-control. In this study, we found a significant positive effect of general self-control on alcohol-specific self-control, but the model only explained 18.8% of the variance in alcohol-specific self-control. This indicates the influence of other factors in addition to the influence of general self-control on alcohol-specific self-control. For example, Spoth et al. (1996) showed that environmental factors such as peers having prosocial norms and parent-child attachment can affect adolescent alcohol refusal skills. A close examination of the predictors of the ability to refrain from drinking alcohol would be helpful in identifying effective means of improving this ability for intervention purposes.

### Strengths and Limitations

The present study is based on a large sample of adolescents and longitudinal data. Due to the longitudinal design it was possible to establish directionality. Furthermore, sophisticated analyses were performed using SEM. Despite these strengths, there are several limitations that should be mentioned. First, alcohol-specific self-control was measured by only two items. Further research should use a validated scale with more items. Second, adolescents’ self-reports were used which could have led to bias, especially related to substance use. Nevertheless, self-reports were found to be a reliable and valid method to measure alcohol use among adolescents (Koning et al., 2010) and other approaches such as using diary reports are hardly feasible in large studies. Third, because of the fact that not all variables of interest were measured at all four time points, the measurement moments that were used for the analysis with digital media use differed. Ideally, analyses were based on the same sample, but priority was given to optimizing the internal validity of the mediational analysis with alcohol use instead of using the same measurement moments. Fourth, this study presents data from 2006 to 2009. The measure of digital media use as used in the current study may be outdated. At the time data collection took place, digital media use was appropriately measured as this was assessed before the rise of smartphones in the Netherlands. However, it can be questioned whether nowadays self-control is similarly strongly related to the frequency of digital media use as to risk behavior such as smoking and drinking alcohol, as it has become normative for adolescents to use their smartphone throughout the whole day (i.e. smartphone use has become an integrated, indispensable part of adolescents’ daily life; Ministerie van Volksgezondheid, Welzijn en Sport, 2019). Therefore, future replications could use a better measurement of digital media use, or replace digital media use by e.g., sexual risk behavior or homework procrastination to test the domain-specificity of alcohol-specific self-control.

## Implications for Prevention Science

In the current study we gained more insight into the relation between general and alcohol-specific self-control and the level of alcohol use among adolescents, and the extent to which alcohol-specific self-control is domain- and substance-specific. The results showed that alcohol-specific self-control fully mediated the effect of general self-control on alcohol use. Due to its critical role, alcohol-specific self-control rather than general self-control may be a target to efficiently reduce the level of alcohol consumption among adolescents through intervention efforts. Interventions can make use of this knowledge by offering training aimed at improving alcohol resistance skills. Interventions aimed at delaying the onset of adolescent alcohol use may, in contrast, target general self-control. Future studies should examine whether general self-control or alcohol-specific self-control is a more proximal predictor of the onset of adolescent alcohol use in order to confirm this assumption. The finding that.

alcohol-specific self-control is domain-, but not substance-specific implies that strengthening adolescents’ ability to refrain from drinking alcohol does have beneficial effects on their ability to resist other substances, but not on their ability to resist temptations in other domains (e.g., digital media use). This has implications for the design of interventions. Since high general self-control can play an important role in reducing multiple adolescent risk behaviors via its effect on domain-specific self-control, interventions aimed at reducing adolescent risk behavior in general may focus on improving general self-control.

## Data Availability

The dataset used and analysed during the current study are available from the corresponding author on reasonable request.
